# Unveiling a Hidden Diaphragmatic Hernia Underlying a Spontaneous Hydropneumothorax: A Diagnostic Surprise

**DOI:** 10.7759/cureus.97175

**Published:** 2025-11-18

**Authors:** Ibrahim Elshafey, Pallavi Setya, Htar Htet Htet Wai, Mohamed Irshadullah Amardeen

**Affiliations:** 1 Emergency Medicine, Acute Medicine, Lincoln County Hospital, United Lincolnshire Teaching Hospitals NHS Trust, Lincoln, GBR; 2 Internal Medicine, Lincoln County Hospital, United Lincolnshire Teaching Hospitals NHS Trust, Lincoln, GBR; 3 Acute Medicine, Lincoln County Hospital, United Lincolnshire Teaching Hospitals NHS Trust, Lincoln, GBR

**Keywords:** abdomen pain, chest tube, chest tube insertion, diaphragmatic hernias, elderly patient management, hydro pneumothorax, non-cardiac chest pain, right-sided pneumothorax, thoracoabdominal injury

## Abstract

Right-sided diaphragmatic hernias in adults are rare and often pose diagnostic challenges due to their atypical clinical and radiological presentations. The coexistence of a spontaneous hydropneumothorax can obscure the underlying pathology, initially diverting attention toward pleural management. We report a rare case of a spontaneous right-sided diaphragmatic hernia discovered incidentally beneath a spontaneous hydropneumothorax in an elderly patient presenting with chest pain, respiratory distress, and abdominal discomfort. Clinical examination revealed decreased breath sounds on the right side with mild abdominal tenderness, and computed tomography of the chest confirmed herniation of abdominal contents into the thoracic cavity, clarifying the dual pathology and guiding definitive surgical management. This case highlights the importance of maintaining a high index of suspicion for diaphragmatic hernia in atypical presentations of hydropneumothorax, emphasizing the role of multimodal imaging, early multidisciplinary collaboration, and adherence to safe chest drain insertion protocols to prevent iatrogenic injury and optimize outcomes.

## Introduction

Diaphragmatic hernias in adults are uncommon and often present significant diagnostic challenges, particularly when occurring on the right side [1,2]. While congenital hernias typically manifest during infancy, delayed presentations may occur in elderly patients, either spontaneously or secondary to factors increasing intra-abdominal pressure [2-4]. Right-sided hernias are especially rare due to the protective effect of the liver, which both obscures diaphragmatic defects and complicates radiographic interpretation [5].

Clinical manifestations are frequently non-specific, ranging from abdominal pain and dyspnoea to features mimicking more common thoracic pathologies, such as pneumothorax or pleural effusion [3,6,7]. Consequently, initial imaging findings may be misinterpreted, leading to inappropriate interventions such as needle decompression or chest drain insertion when herniated abdominal contents are present [3,5,6].

We report a rare case of an 82-year-old woman presenting with chest pain, dyspnoea, and abdominal discomfort, in whom a spontaneous right-sided diaphragmatic hernia was concealed beneath a spontaneous hydropneumothorax. The condition was initially misdiagnosed as a simple pneumothorax with effusion, prompting chest drain insertion. The hernia was subsequently identified on computed tomography (CT), underscoring the importance of maintaining a high index of suspicion and pursuing advanced imaging when clinical and radiographic findings are discordant [3,5,7].

## Case presentation

An 82-year-old woman presented to the Emergency Department with acute right-sided chest and upper abdominal pain that began the previous evening while she was seated. The pain was continuous, radiating to the right shoulder, and associated with nausea and reduced appetite. Her medical history included hypertension and dyslipidaemia, with no recent trauma or prior abdominal surgery.

On examination, her vital signs were as follows: heart rate of 94 bpm, respiratory rate of 20 breaths/min, blood pressure of 134/80 mmHg, temperature of 36.3°C, and oxygen saturation of 96% on room air. Clinical assessment revealed decreased air entry over the right hemithorax and tenderness in the right upper quadrant. Shortly after arrival, her oxygen saturation dropped to 80% on room air, improving to 89% with high-flow oxygen at 10 L/min.

**Figure 1:**
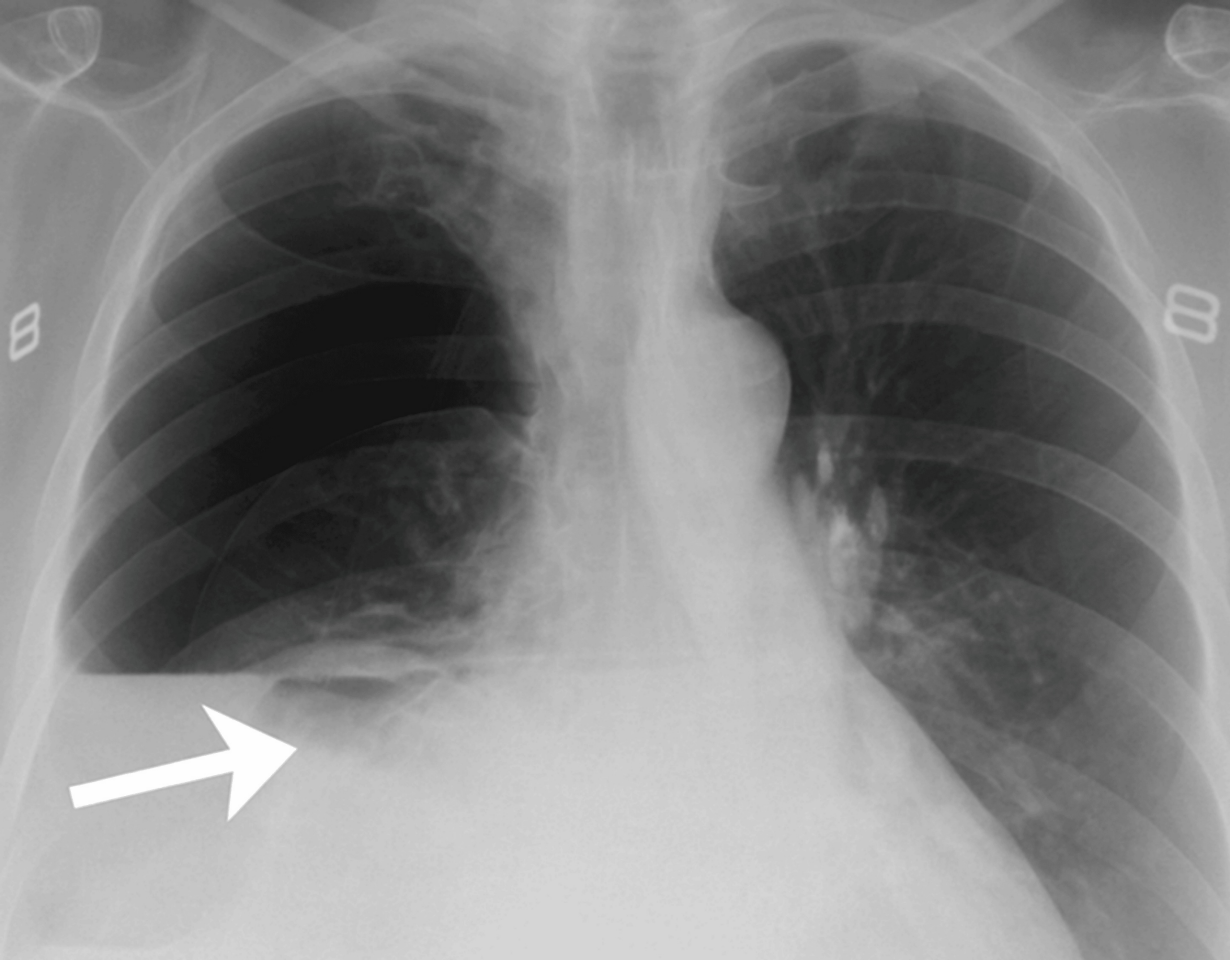
Chest X-ray (frontal view) showing a large right-sided radiolucent area with a visible air-fluid level, suggestive of hydropneumothorax. There is significant opacity occupying the lower right hemithorax, with a sharp horizontal margin indicating a fluid level. The left lung appears fully expanded. A prominent arrow highlights a region near the right hemidiaphragm, where subtle curvilinear densities—possibly representing bowel loops—are faintly visible but were initially overlooked. This image was misinterpreted as a large hydropneumothorax, leading to chest drain insertion before a CT scan confirmed the actual diagnosis of a right-sided diaphragmatic hernia.

A chest X-ray demonstrated a right-sided pneumothorax with an associated pleural effusion and elevation of the right hemidiaphragm (Figure 1). No bowel loops were identified at that stage. Due to worsening hypoxia and clinical deterioration, needle decompression was attempted but proved unsuccessful. A 12-French intercostal chest drain was subsequently inserted under aseptic conditions, resulting in improvement of her oxygen saturation to 94-95% on 15 L oxygen and partial relief of her pain.

**Figure 2:**
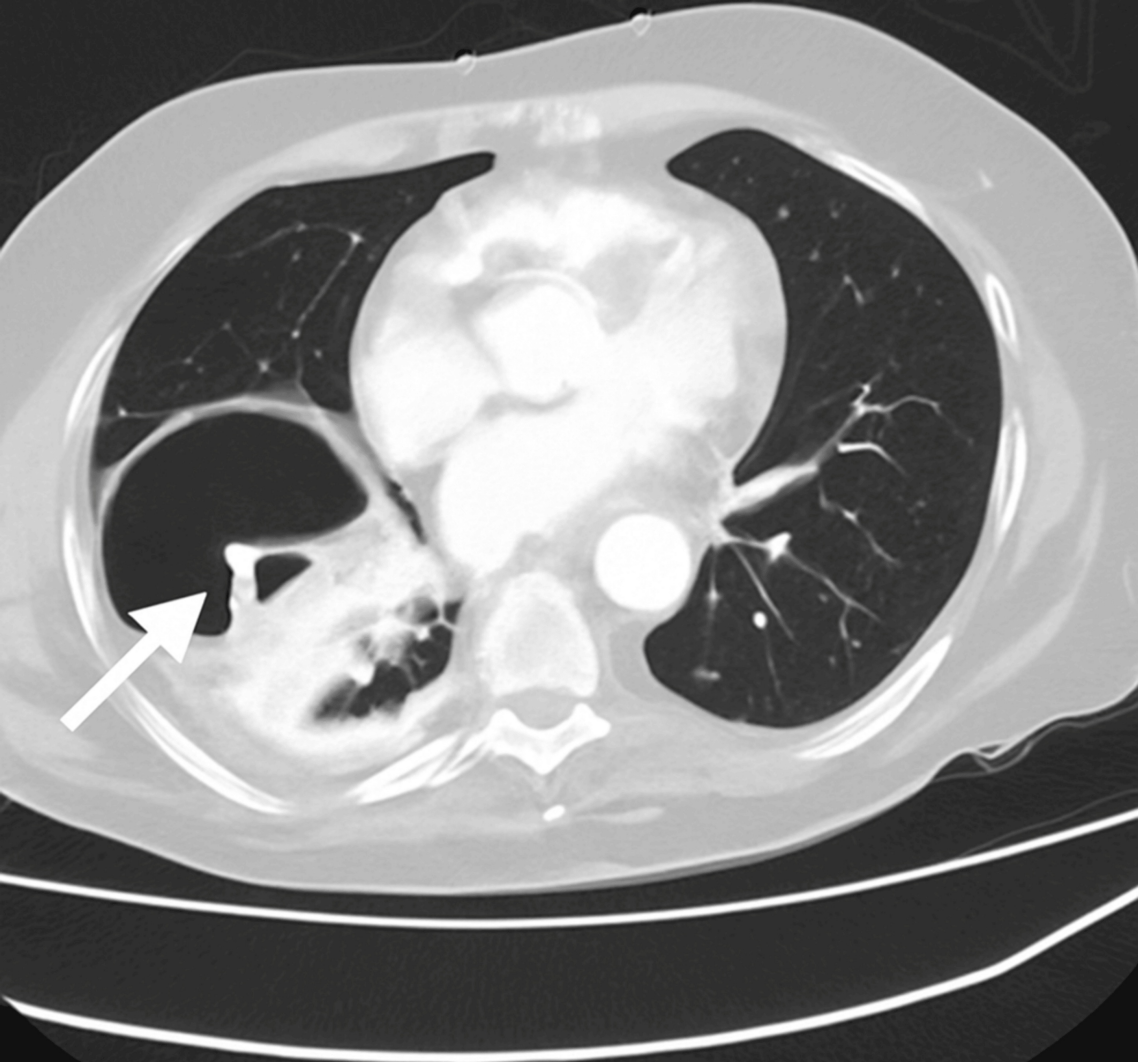
Axial CT scan of the chest revealing a large right-sided intrathoracic air-filled structure with internal soft tissue density, consistent with herniated bowel loops. The bowel wall and mucosal folds are clearly visible within the thoracic cavity, delineating the presence of abdominal contents that have passed through a diaphragmatic defect. The arrow highlights this sharply marginated structure—representative of a segment of small intestine—displacing adjacent lung parenchyma and confirming the diagnosis of a right-sided diaphragmatic hernia. This CT scan was obtained after chest drain placement and played a pivotal role in correcting the initial misdiagnosis of hydropneumothorax.

To further evaluate the underlying cause, a contrast-enhanced CT scan of the chest and abdomen was performed. The CT revealed a right-sided diaphragmatic hernia with herniation of small bowel loops into the thoracic cavity, without evidence of bowel compromise or perforation (Figure 2).

The case was initially discussed with the thoracic surgery team and later referred to general surgery for definitive management. The patient underwent successful surgical repair of the diaphragmatic defect. Intraoperative findings confirmed the presence of intact small bowel loops within the right thoracic cavity. Her postoperative recovery was uneventful, and she was discharged with outpatient surgical follow-up.

## Discussion

Right-sided diaphragmatic hernias in adults are exceptionally rare, especially among the elderly, and they pose significant diagnostic challenges [2,4]. Approximately 80-90% of diaphragmatic hernias occur on the left side, since the right hemidiaphragm is protected by the liver and its embryologic closure occurs earlier, making right-sided herniation much less common [4]. Clinical presentations in adults are often atypical, and overlapping thoracic imaging findings can easily lead to misdiagnosis [3,4]. Consequently, a diaphragmatic hernia on the right may masquerade on chest radiographs as more prevalent conditions - for instance, herniated abdominal organs in the right thorax can mimic a large pneumothorax or pleural effusion on X-ray [3]. This diagnostic pitfall has been documented in the literature and can mislead clinicians away from the correct diagnosis. While many diaphragmatic hernias remain asymptomatic until discovered incidentally, in some cases, they can lead to life-threatening complications, such as bowel strangulation, intrathoracic organ perforation, or severe respiratory compromise if not promptly recognized [2].

In our case, the initial chest radiograph (Figure 1) was interpreted as a massive right-sided hydropneumothorax, prompting the insertion of a chest drain. However, a subsequent CT scan (Figure 2) clearly demonstrated the presence of intrathoracic bowel loops, revealing a right-sided diaphragmatic hernia. This scenario mirrors other reported cases in which diaphragmatic hernias were initially mistaken for pleural fluid collections or pneumothorax on imaging [3]. In several such cases, the error was only recognized after an invasive procedure had already been performed-for example, chest drain placements that resulted in iatrogenic injuries such as intestinal laceration or even gastric perforation due to the unrecognized herniated viscus in the chest [7,8]. This case adds to the limited literature on right-sided diaphragmatic hernias in elderly patients and highlights the risk of iatrogenic injury from chest drain insertion in misdiagnosed cases. To the best of our knowledge, there are no previously published reports of a right-sided diaphragmatic hernia in an elderly patient radiologically mimicking hydropneumothorax, which underscores the clinical relevance of this presentation.

Delayed presentations of congenital diaphragmatic hernias in older adults are thought to arise from the gradual enlargement of an unrecognized small defect (or a tiny tear) in the diaphragm over time. Various factors that increase intra-abdominal pressure - such as heavy lifting, chronic coughing, repeated vomiting, or even forceful sneezing - can precipitate an acute herniation once the diaphragmatic defect widens sufficiently [4]. Minor trauma in the remote past has also been implicated as a possible precipitant in some “spontaneous” hernias of adulthood [4]. Additionally, age-related changes in muscle and connective tissue may weaken the diaphragm’s integrity; patients over 50 years old could therefore be at higher risk for such late-onset herniations [4]. The clinical manifestations in adults are notoriously variable and nonspecific, ranging from respiratory symptoms (dyspnea, chest or shoulder pain) to gastrointestinal complaints (abdominal distension, nausea, vomiting) [4]. Because these symptoms often mimic far more common cardiopulmonary or gastrointestinal conditions, clinicians must maintain a high index of suspicion when faced with an atypical presentation [4]. In particular, late-presenting congenital diaphragmatic hernias may be misinterpreted on imaging as pleural effusions or pneumothorax, delaying appropriate treatment [9]. If unrecognized, a diaphragmatic hernia can rapidly progress to critical complications as noted above, so early consideration of this diagnosis is crucial [2,5].

Imaging is the cornerstone of diagnosis, and CT is widely regarded as the definitive modality for confirming diaphragmatic hernias in adults [4]. Chest X-ray is a useful initial investigation but has limitations: plain radiographs can fail to detect a significant portion of diaphragmatic hernias or misrepresent them as other pathologies [3]. By contrast, CT offers superior sensitivity and provides clear delineation of any herniated viscera and the diaphragmatic defect, facilitating an accurate diagnosis [3,4]. In an elderly patient, if the chest X-ray findings do not correlate with the clinical picture, it is prudent to obtain an early CT scan before proceeding with invasive interventions such as chest tube placement [3]. Adopting a careful, multidisciplinary approach - involving emergency physicians, radiologists, and surgeons - is essential to avoid unnecessary procedures that could cause harm. Prompt specialist consultation and cross-sectional imaging help ensure that the patient receives the appropriate management (typically surgical repair of the diaphragm) without incurring iatrogenic injury.

This case underscores the importance of considering diaphragmatic hernia as a rare yet critical mimic of hydropneumothorax. Heightened awareness, early CT confirmation, and team-based decision-making can prevent unnecessary procedures and improve outcomes.

## Conclusions

This case illustrates the diagnostic complexity of right-sided diaphragmatic hernias in elderly patients, especially when obscured by a concurrent spontaneous hydropneumothorax. Clinicians should maintain a high index of suspicion when chest and abdominal symptoms coexist, even if initial imaging suggests a pneumothorax, as early CT evaluation can reveal dual pathologies and guide appropriate surgical management.

Procedural vigilance during chest drain insertion is essential to prevent iatrogenic injury in patients with unrecognized diaphragmatic defects or herniated abdominal contents. A multidisciplinary approach that integrates clinical assessment, imaging correlation, and surgical input is vital to ensure timely diagnosis and optimize patient outcomes.
